# Supplementary figures and images for: Detection of terrestrial mammals using environmental DNA during heavy rainfall events and associated influencing factors
Source: PeerJ. 2025 Oct 15;13:e20166. doi: 10.7717/peerj.20166 (PMC12535231; doi:10.7717/peerj.20166)

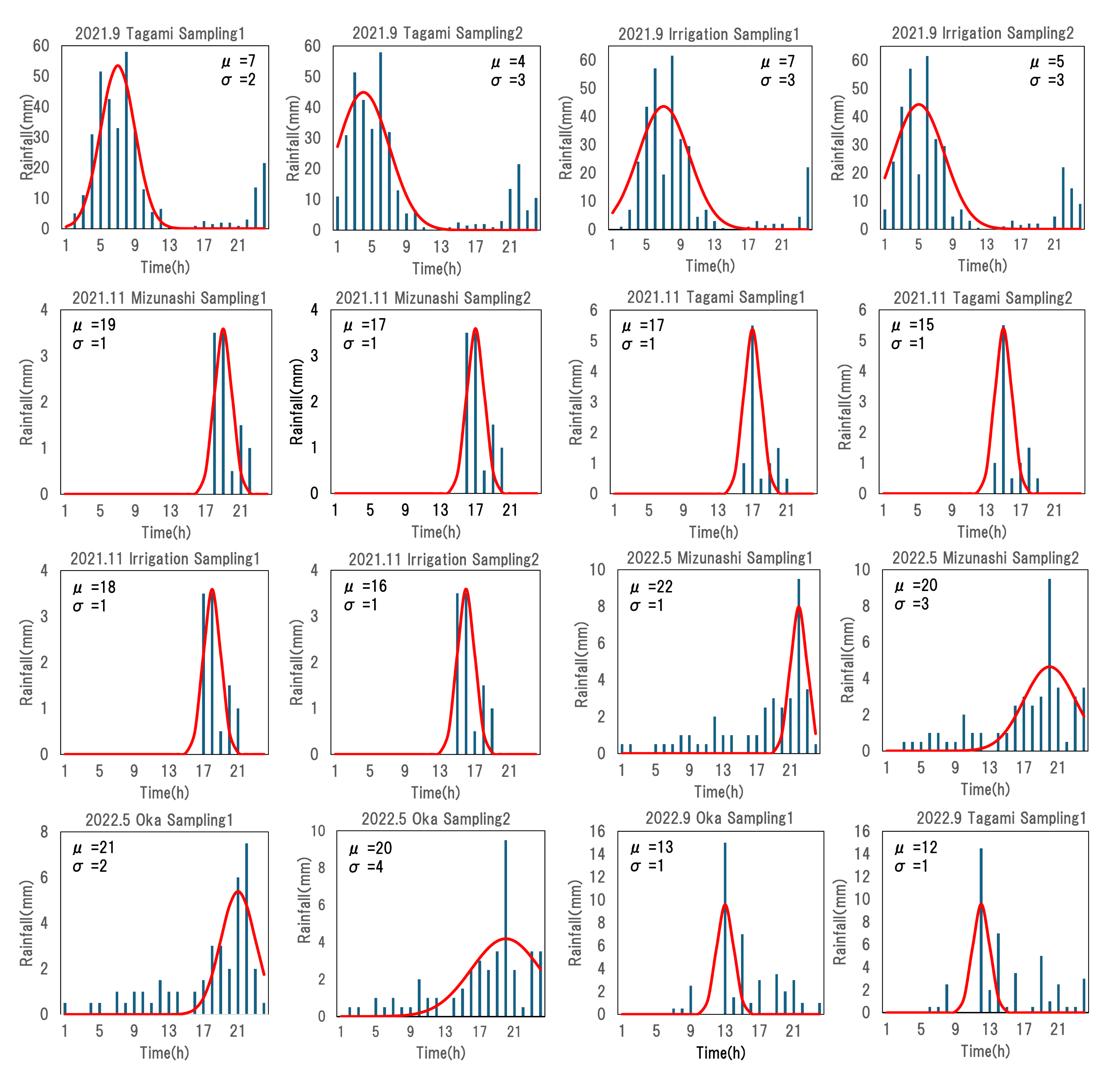

Supplement: Supplemental Information 1 — The “Sampling1” and “Sampling2” indicate the first and second sampling of the day, respectively. The bar chart represents the rainfall amounts, while the red curve represents the normal distribution. “σ” and “μ” represent the parameters of normal distribution. The RMSE value represents the root mean square error between the observed rainfall and the calculated rainfa ll. [file peerj-13-20166-s001.png]

Prime=450 $\mu$ M, probe=125 $\mu$ M

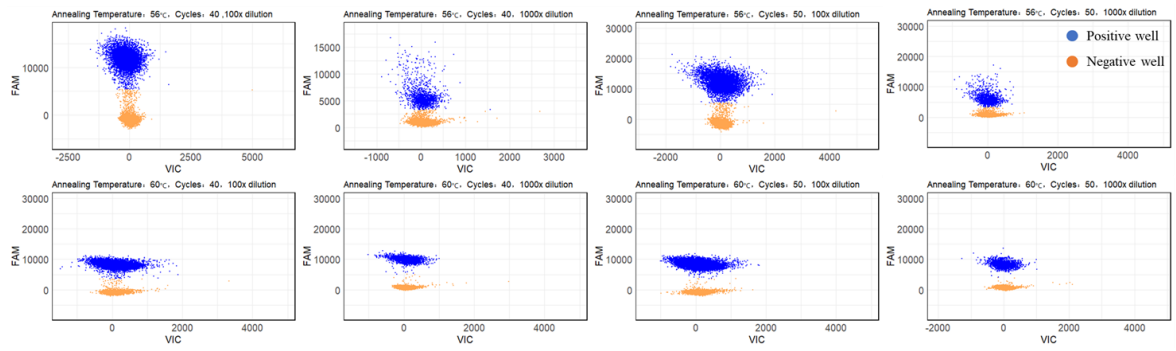

Prime=900 $\mu$ M, probe=125 $\mu$ M

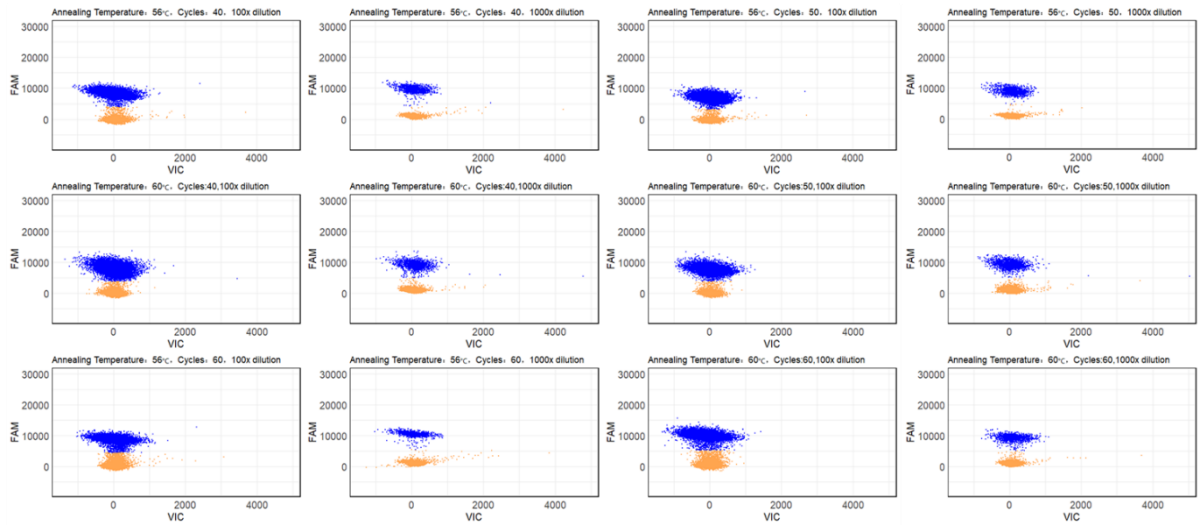

Prime=900 $\mu$ M, probe=250 $\mu$ M

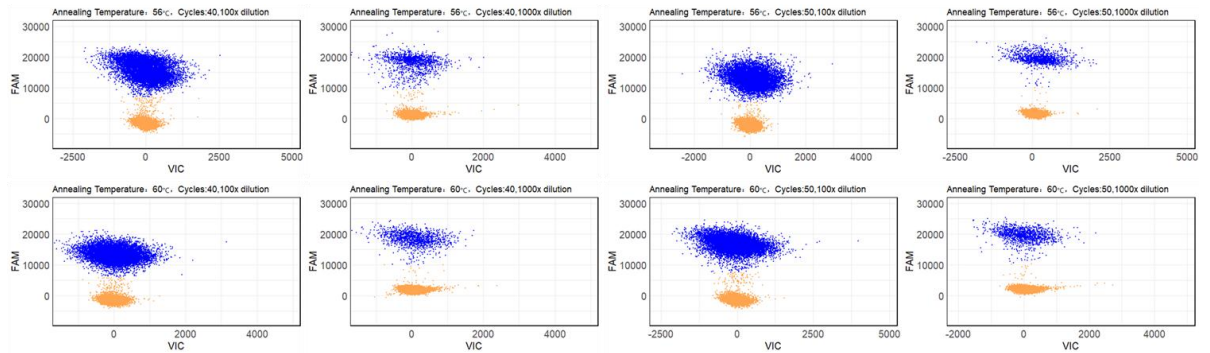

Supplement: Supplemental Information 2 — Blue points represent positive wells, while orange points represent negative wells. [file peerj-13-20166-s002.pdf]

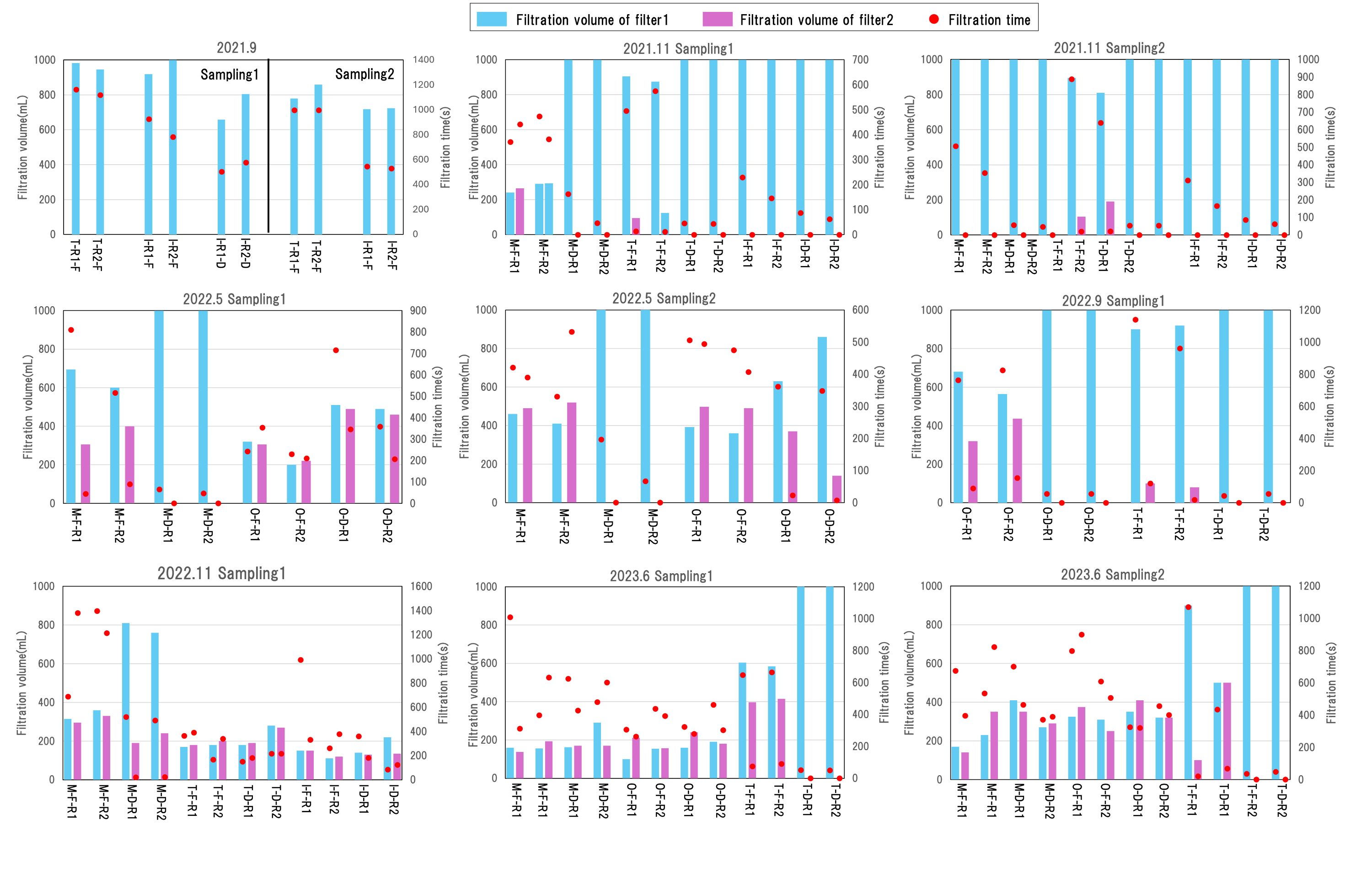

Supplement: Supplemental Information 3 — The “Sampling1” and “Sampling2” indicate the first and second sampling of the day, respectively. “M”, “O”, “T”, and “I” represent the Mizunashi River, Oka River, Tagami River, and irrigation canal. “F” indicates results of a glass fiber filter with a pore size of 0.7 μm while “D” indicates a glass fiber filter with a pore size of 2.7 μm. “R1” and “R2” indicate replicate1 and replicate2. The samples from September 2021 only recorded total filtration volume and filtration time. [file peerj-13-20166-s003.png]
